# Rigorous Guidelines Deserve to Be Read: WHO New Guidance on Obesity in Children and Adolescents, and Our First Guideline Summary

**DOI:** 10.1002/gin2.70095

**Published:** 2026-10-27

**Authors:** Ivan D. Florez

**Affiliations:** ^1^ Department of Pediatrics University of Antioquia Medellin Colombia; ^2^ Pediatric Intensive Care Unit Clinica Las Américas‐AUNA Medellin Colombia

Obesity is a global public health crisis, affecting more than one billion people worldwide. When it begins in childhood or adolescence, the problem is even larger: it shapes health, opportunity, and stigma for the rest of a young person's life. In 2022, more than 390 million children and adolescents aged 5–19 years were living with overweight, including over 160 million with obesity, a prevalence that has quadrupled since 1990 [1, 2]. In 2024 alone, 35 million children under five were overweight [3]. The rise is now steepest in low‐ and middle‐income countries, often alongside persisting undernutrition [2]. The consequences are not deferred. Early‐life obesity brings earlier type 2 diabetes and cardiovascular disease, psychosocial harm and stigma, and tracks strongly into adulthood [2].

Confronting this burden requires trustworthy guidance on what to do. Guidance on childhood obesity has proliferated, yet much of it was written for high‐income settings, with little attention to equity, obesogenic environments, or delivery through primary care [4]. The stakes are even higher when the developer is the World Health Organisation (WHO). Ministries of health adopt and adapt WHO guidelines, and they reach populations that no professional society guideline will. The two complementary WHO guidelines on the integrated management of obesity in children and adolescents, launched on 7 October 2026, fill a long‐standing gap [5]. Previous WHO guidance focused on prevention; this addresses the care of children and adolescents already living with obesity.

What makes a guideline trustworthy is its methods [6]. The WHO handbook for guideline development requires that recommendations rest on systematic reviews, and that the guideline group not only assess the certainty of the evidence but also weigh benefits and harms, values and preferences, resource use, equity, acceptability and feasibility [7]. That transparent, rigorous process separates expert opinion from recommendations that clinicians, programmes, and ministries can act on with confidence.

In this issue of Clinical and Public Health Guidelines, García‐Casal and Pardo‐Hernández provide a summary of the new WHO guidelines [8]. Full guidelines are long, and their recommendations are easily lost on the people who need them most. A structured summary presents the scope, the key recommendations with their strength and certainty of evidence, and the main implementation considerations, so that readers can quickly see what is new and what applies in their own setting. Publishing it open access in a journal read by the guideline community extends that reach well beyond the WHO website. This is the commitment at the heart of CPHG. As the official journal of the Guidelines International Network, we exist to make rigorous, evidence‐based recommendations visible, understandable and usable.

We intend this summary to be the first of many. Many organisations produce guidelines to high methodological standards that still reach only a fraction of their intended audience. CPHG will publish concise, structured summaries of rigorously developed guidelines from organisations around the world, to increase their visibility, support their adoption and adaptation, and strengthen the link between those who develop recommendations and those who act on them. We invite our readers to read and share the summary of WHO's new guidance on obesity in children and adolescents. We also invite guideline developers who want a wider audience for their evidence‐based recommendations to work with us on further guideline summaries.

## Author Contributions

**Ivan D. Florez:** conceptualisation, writing – original draft, writing – review and editing.

## Conflicts of Interest

The author declares no conflicts of interest.

## Data Availability

The author has nothing to report.
